# Comparative genomics of drug-resistant strains of *Mycobacterium tuberculosis* in Ecuador

**DOI:** 10.1186/s12864-022-09042-1

**Published:** 2022-12-21

**Authors:** Gabriel Morey-León, Derly Andrade-Molina, Juan Carlos Fernández-Cadena, Luisa Berná

**Affiliations:** 1 Laboratorio de Interacciones Hospedero-Patógeno, Unidad de Biología Molecular, Institut Pasteur de Montevideo, Montevideo, Uruguay; 2 Universidad de Guayaquil, Guayaquil, Ecuador; 3 Facultad de Ciencias de la Salud, Universidad Espíritu Santo, Samborondón, Ecuador; 4 Laboratorio de Ciencias Ómicas, Universidad Espíritu Santo, Samborondón, Ecuador; 5 Facultad de Ciencias, Unidad de Genómica Evolutiva, Universidad de La República, Montevideo, Uruguay

**Keywords:** Tuberculosis, Drug-resistance, Lineages, Ecuador, Pan Genome

## Abstract

**Background:**

Tuberculosis is a serious infectious disease affecting millions of people. In spite of efforts to reduce the disease, increasing antibiotic resistance has contributed to persist in the top 10 causes of death worldwide. In fact, the increased cases of multi (MDR) and extreme drug resistance (XDR) worldwide remains the main challenge for tuberculosis control. Whole genome sequencing is a powerful tool for predicting drug resistance-related variants, studying lineages, tracking transmission, and defining outbreaks. This study presents the identification and characterization of resistant clinical isolates of *Mycobacterium tuberculosis* including a phylogenetic and molecular resistance profile study by sequencing the complete genome of 24 strains from different provinces of Ecuador.

**Results:**

Genomic sequencing was used to identify the variants causing resistance. A total of 15/21 isolates were identified as MDR, 4/21 as pre-XDR and 2/21 as XDR, with three isolates discarded due to low quality; the main sub-lineage was LAM (61.9%) and Haarlem (19%) but clades X, T and S were identified. Of the six pre-XDR and XDR strains, it is noteworthy that five come from females; four come from the LAM sub-lineage and two correspond to the X-class sub-lineage. A core genome of 3,750 genes, distributed in 295 subsystems, was determined. Among these, 64 proteins related to virulence and implicated in the pathogenicity of *M. tuberculosis* and 66 possible pharmacological targets stand out.

Most variants result in nonsynonymous amino acid changes and the most frequent genotypes were identified as conferring resistance to rifampicin, isoniazid, ethambutol, para-aminosalicylic acid and streptomycin. However, an increase in the resistance to fluoroquinolones was detected.

**Conclusion:**

This work shows for the first time the variability of circulating resistant strains between men and women in Ecuador, highlighting the usefulness of genomic sequencing for the identification of emerging resistance. In this regard, we found an increase in fluoroquinolone resistance. Further sampling effort is needed to determine the total variability and associations with the metadata obtained to generate better health policies.

**Supplementary Information:**

The online version contains supplementary material available at 10.1186/s12864-022-09042-1.

## Introduction

Tuberculosis (TB), caused by *Mycobacterium tuberculosis* (Mtb), is one of the top 10 causes of death in 2020. The World Health Organization estimated that 9.9 million people were infected, and 1.5 million died [1]. Despite constant efforts over the years to lessen the impact of this disease, several challenges, such as patient adherence, long duration of treatment, and late diagnosis, have reduced the effectiveness of TB therapy [2]. In addition, the frequent emergence of drug-resistant strains is a serious global threat and poses significant challenges to public health, especially in low- and middle-income countries.

Drug resistance in bacteria, particularly in *M. tuberculosis*, negatively affects health programs worldwide due to the increase of resistant strains that cannot be treated with existing anti-TB drug therapy. Indeed, tuberculosis is considered to be one of the "Big three" infectious diseases worldwide [3]. Patients with resistant TB may develop it due to clinical factors such as late or inappropriate diagnosis, ineffective treatment, poor compliance to the regimen, and exposure to circulating multidrug-resistant and extensively drug-resistant tuberculosis (MDR/XDR-TB) strains [2]. The slow growth of Mtb slows diagnosis and consequently limits the timely detection of resistance to anti-TB drugs. This contributes to the rising incidence of MDR and XDR TB worldwide. Conversely, the accumulation of point mutations in coding regions for drug targets or drug-converting enzymes is an essential mechanism for acquiring resistance in Mtb [4].

The *Mycobacterium tuberculosis* complex is composed of the human-adapted members, the *Mycobacterium tuberculosis *sensu stricto (lineages 1, 2, 3, 4, and 7) and *M. tuberculosis var. africanum* (lineages 5 and 6; *M. africanum*), distinct phylogenetic lineages that have evolved over millennia [5–7] and lineage 8 and 9 are new lineages recently discovered in Africa [8]. Research on the distribution of lineages of Mtb in South America has shown that the Euro-American lineage (lineage 4) has a variable distribution of lineages/sub-lineages between and within these countries, establishing that the predominant strains in their America have evolved from the Euro-American lineage [9]. This lineage has been described as more transmissible than others [10]. From 10 sub-lineages, including in Lineage 4, LAM (L4.3), Haarlem (L4.1.2), X-type and T families are the most observed members of the Euro-American lineage in South and Central America and the Caribbean, a distribution profile shared with Europe and Middle Africa [11–14].

In 2015, due to drug resistance rising worldwide, the WHO proposed expanding rapid testing and detection of cases as one of the five high-priority actions to tackle the global DR-TB crisis [15]. As part of these actions, some molecular biology-based diagnostic methods have been applied in a clinical setting for their versatility and speed, including variants of Xpert MTB/RIF and GenoType MTBDRplus [16–19]. Although these methods are rapid and straightforward, Mtb continuously evolves through the genomic acquisition of single nucleotide polymorphisms (SNPs), insertions, and deletions (indels), impacting the ability to discriminate strains without the classic regions used for resistance detection [20]. Whole-genome sequencing (WGS), as a molecular diagnostic tool, has been greatly developed in TB research and is clinically useful for predicting drug resistance, lineage, tracing transmission, and defining outbreaks [21–26]. Furthermore, WGS has the potential to determine drug resistance much faster than traditional phenotypic drug susceptibility testing (DST) [27] and does not require biological safety infrastructure [28, 29]. These technologies are used differentially, with the highest concentration of sequenced strains found in 16 countries [30–32]. However, for some countries, particularly in South America, WGS information on the variability and distribution of circulating strains is unavailable.

In Latin America, are extensive information on *Mycobacterium tuberculosis,* highlighting its genetic diversity analyzed by molecular methods*; despite* this, very few studies have been carried out in Ecuador, in which the LAM and Haarlem lineages are described as the more prevalent, and a little for the Beijing family [33–35]. Recently Garzon-Chavez et al. [36] determined that the main circulating lineages in Ecuador were LAM, Haarlem, and S and only applied WGS for resolving the classification in 8 isolates with an ambiguous result from 373 strains assessed by 24 loci-MIRU-VNTR and DR analysis, previously. In this sense, it is essential to know the genetic composition of *M. tuberculosis*, especially the variations associated with drug resistance distributed within its genome. This study aims to assess the lineage and molecular resistance profile characterization in clinical isolates of *M. tuberculosis* through whole-genome sequencing.

## Results

In order to assess the genetic variability of resistant M. tuberculosis strains, twenty-four culture-confirmed clinical isolates of M. tuberculosis with at least one antibiotic resistance were selected for whole genome sequencing. From them, the bacillary load test in the microscopy smear was greater than two crosses, and 66,7% of patients relapsed. The isolates come from four regions of western Ecuador (20, 83.3%, Guayaquil; 2, 8.3%, El Empalme, 1, 4.2% Babahoyo and Chone, respectively). The majority of the patients were female (64,5%); the mean age was 42.9 ± 12.9 years; comorbidity like HIV was present in three patients (Table 1). All isolates were only tested for phenotypic resistance to four first-line drugs: rifampicin (R), isoniazid (H), ethambutol (E), and pyrazinamide (Z); and one second-line drug: streptomycin (S). Thus, a wide range of drug resistance profiles were covered: 85.7% are polydrug resistant (50% resistant to all first-line drugs plus streptomycin), and 14.3% are MDR (Table 1 and Supplementary table 1).

**Table 1:** Information on isolates and genomic statistics for analyzed samlples

Clinical isolate	Age	Phenotypic resistance profile	Phenotypic classification according to WHO	Coverage	Contigs	Assembly size (Mb)	CDS	tRNA	Total Mutations
S001_MTb_EC	49	H-R-S	Poly-resistance	35X	144	4,4	4.043	52	1.460
S007_MTb_EC	53	H-R	MDR	33X	152	4,3	4.086	53	1.357
S008_MTb_EC	53	H-R	MDR	41 X	149	4,3	4.054	53	1.378
S013_MTb_EC	34	H-R-S-E-Z	Poly-resistance	34X	159	4,3	4.050	53	1.206
S017_MTb_EC	34	H-R-S	Poly-resistance	33X	141	4,4	4.116	54	1.428
S022_MTb_EC	38	H-R-S-E-Z	Poly-resistance	41X	144	4,4	4.116	54	1.222
S036_MTb_EC	27	H-R-S-Z	Poly-resistance	38X	146	4,3	4.021	52	1.306
S039_MTb_EC	26	H-R-S	Poly-resistance	38X	150	4,3	4.051	53	1.430
S040_MTb_EC	29	H-R-Z	Poly-resistance	33X	162	4,3	4.056	53	1.230
S046_MTb_EC	71	H-R-S	Poly-resistance	38X	150	4,3	4.058	53	1.377
S059_MTb_EC	58	H-R-E	Poly-resistance	47X	190	4,3	4.055	52	1.224
S066_MTb_EC	70	H-R-S-Z	Poly-resistance	38X	150	4,3	4.056	53	853
S070_MTb_EC	36	H-R	MDR	36X	144	4,3	4.097	53	1.209
S091_MTb_EC	40	H-R-S-E-Z	Poly-resistance	54X	137	4,3	4.072	53	1.292
S149_MTb_EC	45	H-R-S-E-Z	Poly-resistance	31X	169	4,3	4.076	58	1.230
S165_MTb_EC	49	H-R-S	Poly-resistance	37X	132	4,3	4.048	52	1.445
S194_MTb_EC	34	H-R-S-E-Z	Poly-resistance	25X	160	4,3	4.060	53	1.182
S196_MTb_EC	28	H-R-S-E-Z	Poly-resistance	29X	138	4,3	4.036	53	1.187
S202_MTb_EC	34	H-R-S-E-Z	Poly-resistance	23X	168	4,3	4.070	53	1.176
S204_MTb_EC	43	H-R-S-E-Z	Poly-resistance	44X	140	4,3	4.094	53	1.326
S205_MTb_EC	45	H-R-S-E-Z	Poly-resistance	26X	159	4,3	4.045	52	1.225

*Abbreviations MDR* Multidrug resistance, *R* rifampicin, *H* isoniazid, *E* ethambutol, *Z* pyrazinamide, *S* streptomycin, *CDS* Coding sequence, *tRNA* transference RNA

**Fig. 1:**
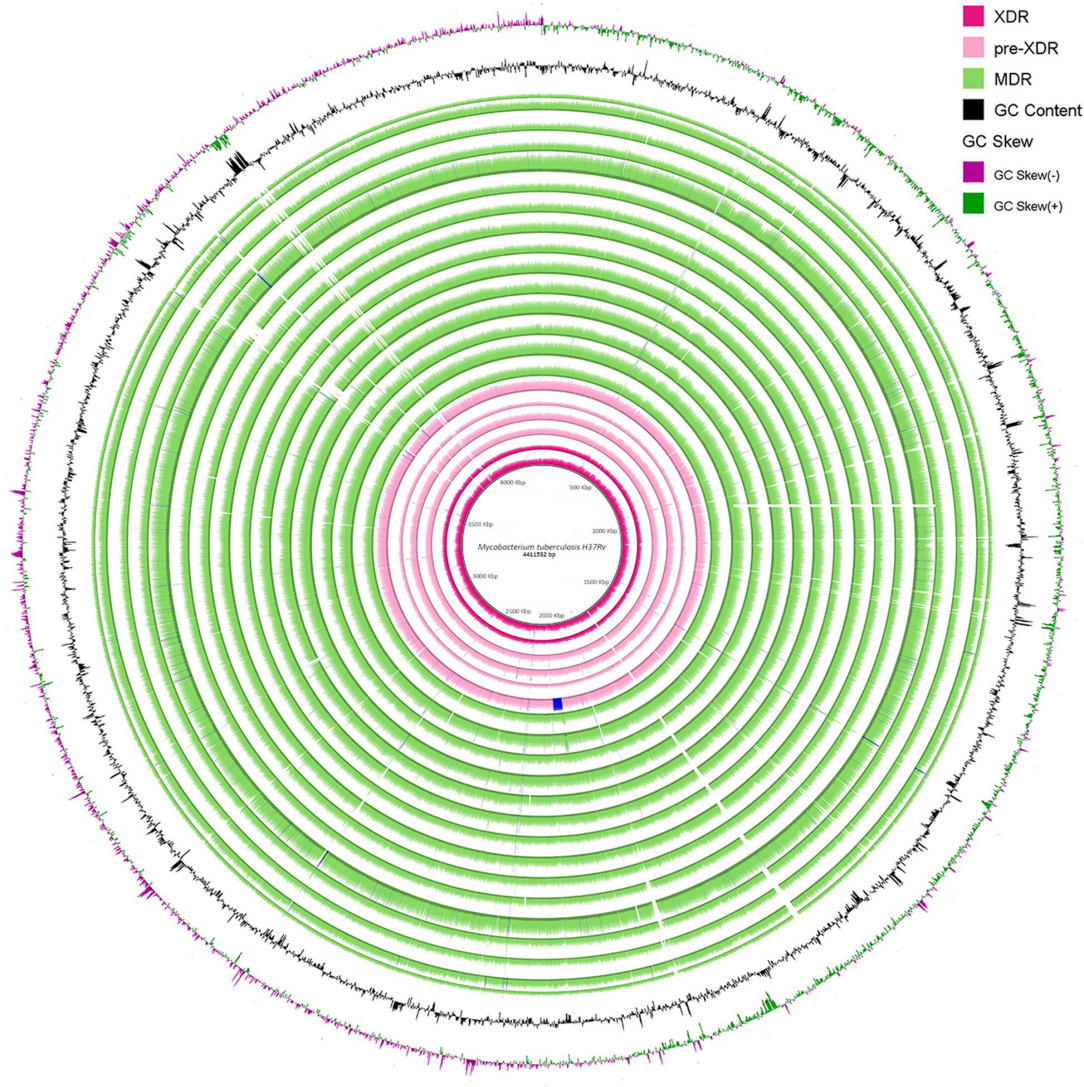
Genome sequence comparison of 21 resistant *M. tuberculosis* genomes (15 MDR, four pre-XDR, and 2 XDR strains) shown by BRIG. Circles display (from the inside): 1. Reference genome of strain H37Rv; 2–21. Read mapping coverage of sequencing reads calculated from 21 genomes (2, 3. XDR strains (turquoise); 4–7. pre-XDR strains (rose) and 8–21 MDR strains (light green)); 23. GC content; 24. GC skew values positive (green) to negative (purple);. The coverage shown in rings 2 through 21 is from mapping the reads against the reference using BWA. The height of the plot in each ring is proportional to the number of reads mapped at each nucleotide position in the reference genome from 0 to 30 × coverage. The lighter area of the rings represents regions with less coverage. Regions with genome coverage greater than 30 × are shown as solid blue bands

The 24 strains isolated were sequenced using the Illumina platform with paired-end reads (2 × 150 bp). The sequences were filtered by the quality and trimmed when necessary. The depth obtained ranged from 22.7X to 53.6X (Table 1). The strains were mapped against the reference *M. tuberculosis* H37Rv (NC_000962.3) (4.4 million base pairs and 4008 protein-coding genes) to verify that they were free of contamination. The sequencing and mapping statistics are presented in Table 1. Most strains have an outstanding quality, depth, and percentage mapping against the reference; however, three strains presented an average depth below 20 and were discarded for further analysis. The 21 remaining strains were *the novo* assembled, and the assemblies obtained were checked for contamination. On average, we recovered draft genomes of 4.3 Mb distributed in 140 contigs per strain with at least an N50 of 56 Kb (Table 1 and Supplementary table 1). Although a complete genome was obtained for all strains, the fragmentation into several contigs is due primarily to regions of extreme base composition and low complexity. This can be seen in Fig. 1, which shows the reference *M. tuberculosis* H37Rv, the percentage of GC and GC-Skew, and the coverage plot of 21 genomes assembled and plotted according to drug resistance profile. Here, common breaks in coverage correspond to peaks in GC or GC-skew.

**Fig. 2:**
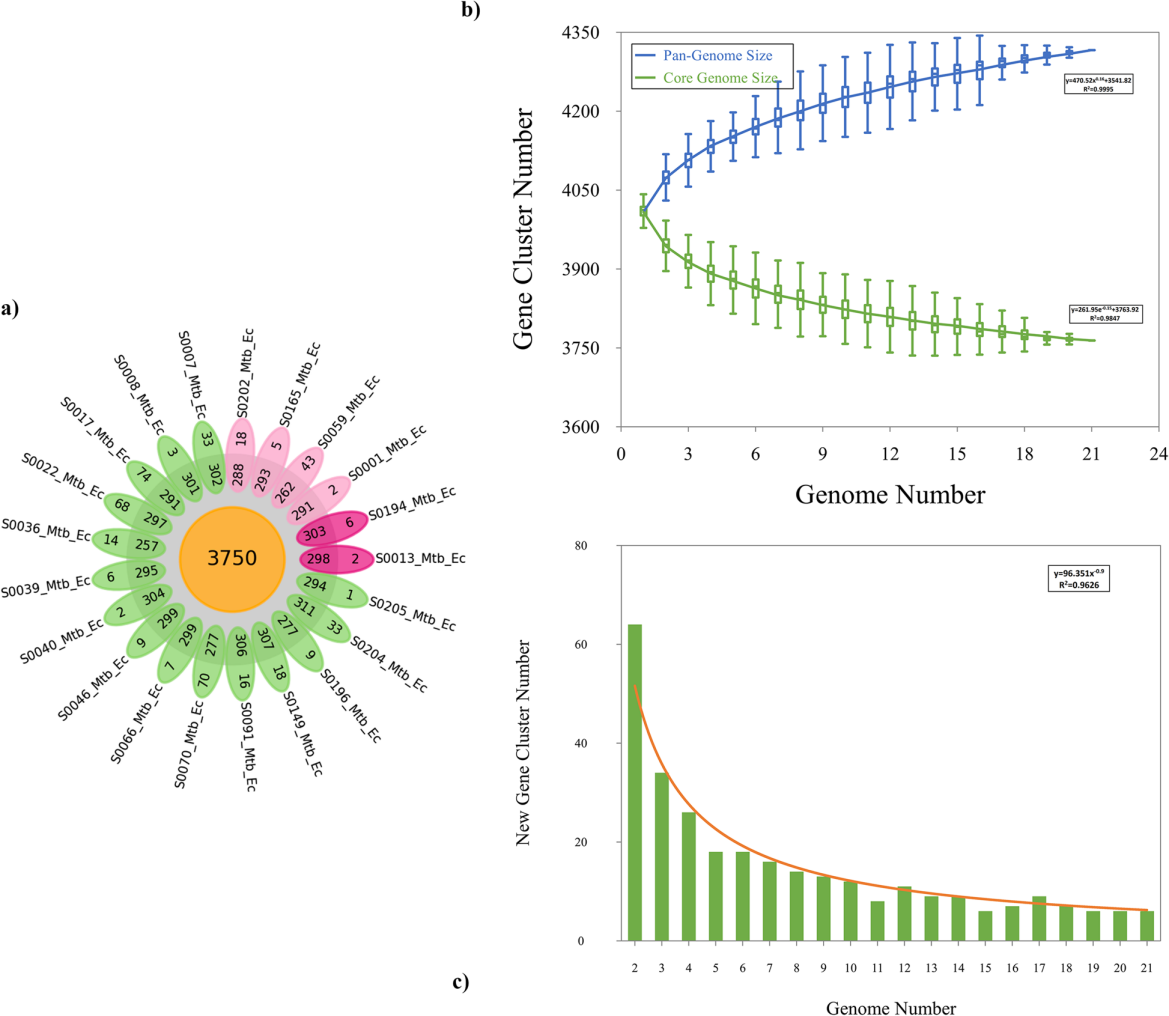
The pangenome of 21 M*. tuberculosis* isolates. **a** Flower plots showing the core, accessory, and strain-specific genes of the 21 Mtb strains. The flower plot shows the core gene number (in the center), the accessory gene number (in the annulus), and the strain-specific gene number (in the petals) for the 21 Mtb strains. **b** Gene accumulation curves of the pangenome (blue) and the core-genome (green). The blue and green boxes denote the Mtb pangenome and core size for each genomic comparison. **c** The curve (red) shows the number of new genes against the increase in the number of Mtb genomes

Prokka and RASTtk were used to determine the structure and function of genes. On average, the Prokka annotation showed the presence of ∼4,119 ± 26.28 genes; among them, 4,065 correspond to coding sequences (CDS), 53 are transfer RNAs, and 1 code for a tmRNAs (Table 1). Based on the annotations, the analysis of the pangenome was carried out with Roary [37]. Thus, the pangenome analysis of 21 genomes of *M. tuberculosis* revealed that there were 4735 gene families defined as the pangenome. The protein-coding genes identified for each strain have slight variation in number, ranging from a minimum of 4021 genes (strain S0036_Mtb_Ec) to a maximum of 4115 genes (strains S0017_Mtb_Ec and S0022_Mtb_Ec). A total of 3750 common genes (core genes) shared by all the genomes analyzed were identified. In addition, 99 soft-core genes (presented in 20 of the 21 strains), 353 accessory genes (shared between two and 19 strains), and 533 cloud genes (strain-specific genes) were identified (Fig. 2A). The core and pangenome size ratio were found to be 0.79; thus, the core forms 79% of the pangenome, representing a rather closed genome with less variability. Gene accumulation was calculated for the core and pangenome. It is observed that after including 21 strains, the increase in the total number of genes is significantly reduced, and on the other hand, the core genome increases minimally, indicating a pangenome close to closing. This can be observed in Figs. 2B and C; from this visualization, it can be suggested that genome characterization of approximately 21 strains provides the genetic repertoire of *M. tuberculosis*.

**Fig. 3:**
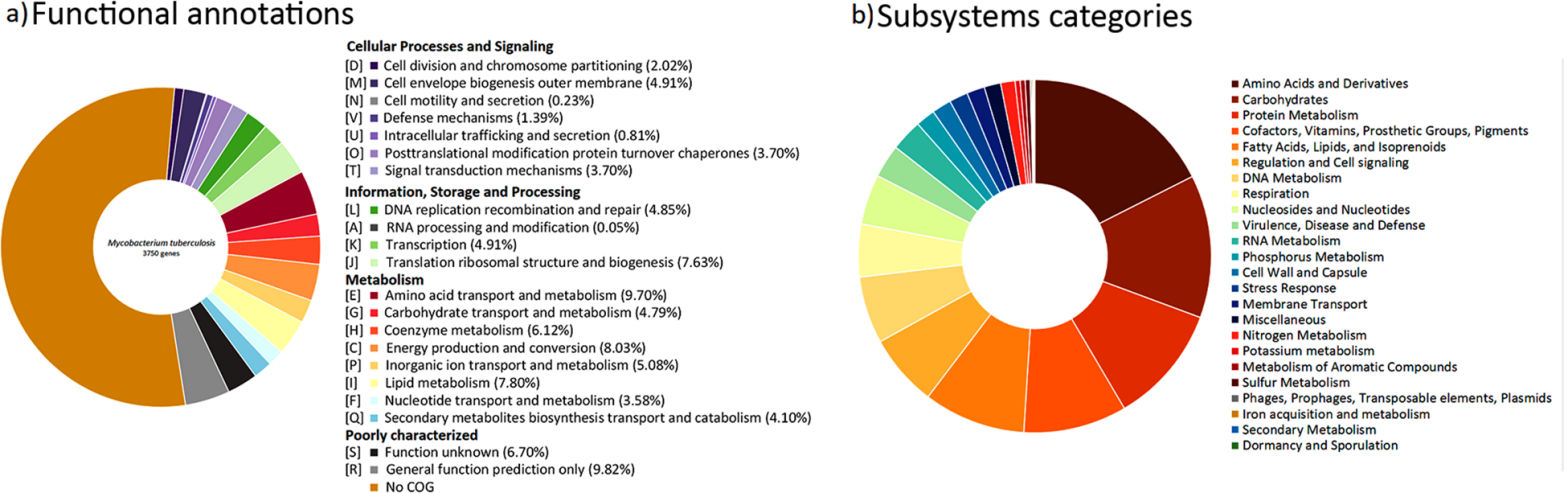
Functional annotation of 21 M*. tuberculosis* genomes. **a** COG categories and subcategories of predicted genes within the core genomes of 21 M*. tuberculosis* genomes by eggNOG Mapper v2.1.7. Each subcategory is graphed as an average percentage of the total number of genes in the core genomes. **b** Subsystem category distribution of 21 M*. tuberculosis* genomes based on RASTtk annotation. Genes analyzed 4,119 ± 26.28; genes in subsystems 1,521 ± 7.60; subsystems identified 295

From the core genes and using Prokka and RASTtk, subsystems were annotated. Using COG annotations, a function was assigned for a mean of 1,731 genes for each strain analyzed. These annotated genes were classified into 21 metabolic pathways within four primary categories, including Metabolism (49.2%); Information, storage, and processing (17.5%); Cellular processes, and signaling (16.8%), and poorly characterized (16.5%). As for many other organisms, almost half of the genes identified in all strains analyzed (~ 2022 genes, ~ 53.9% of the total) could not be assigned function (Supplementary table 2 and Fig. 3a).

In addition, using RASTtk [38], ∼1,521 genes were assigned to the subsystems (a group of proteins that implement a specific biological process or structural complex). It was observed that almost 18% of the annotated protein-coding genes were associated with amino acids and their derivatives (267 ± 3.04), followed by genes related to carbohydrates (13.1%, 199 ± 1.74), protein metabolism (10.9%, 166 ± 0.68), cofactors, vitamins, prosthetic groups, and pigments (9.5%, 144 ± 4.65 genes). About 60% of the genes identified in subsystems are involved in the Metabolism of proteins, amino acids, carbohydrates, lipids, and cofactors (Fig. 3b). Cell regulation and signaling (6.6%, 101 ± 1.7) and DNA metabolism (6.1%, 93 ± 1.32) also appear as subsystems with a relevant role. Partly due to the number of strains, a higher diversity in the percentage of genes in the subsystems was found for MDR strains than for pre-XDR and XDR strains (Supplementary table 3). Most of the genes involved in amino acid biosynthesis were conserved and proved to have an essential function for pathogenicity in bacteria, including *M. tuberculosis* [39].

**Fig. 4:**
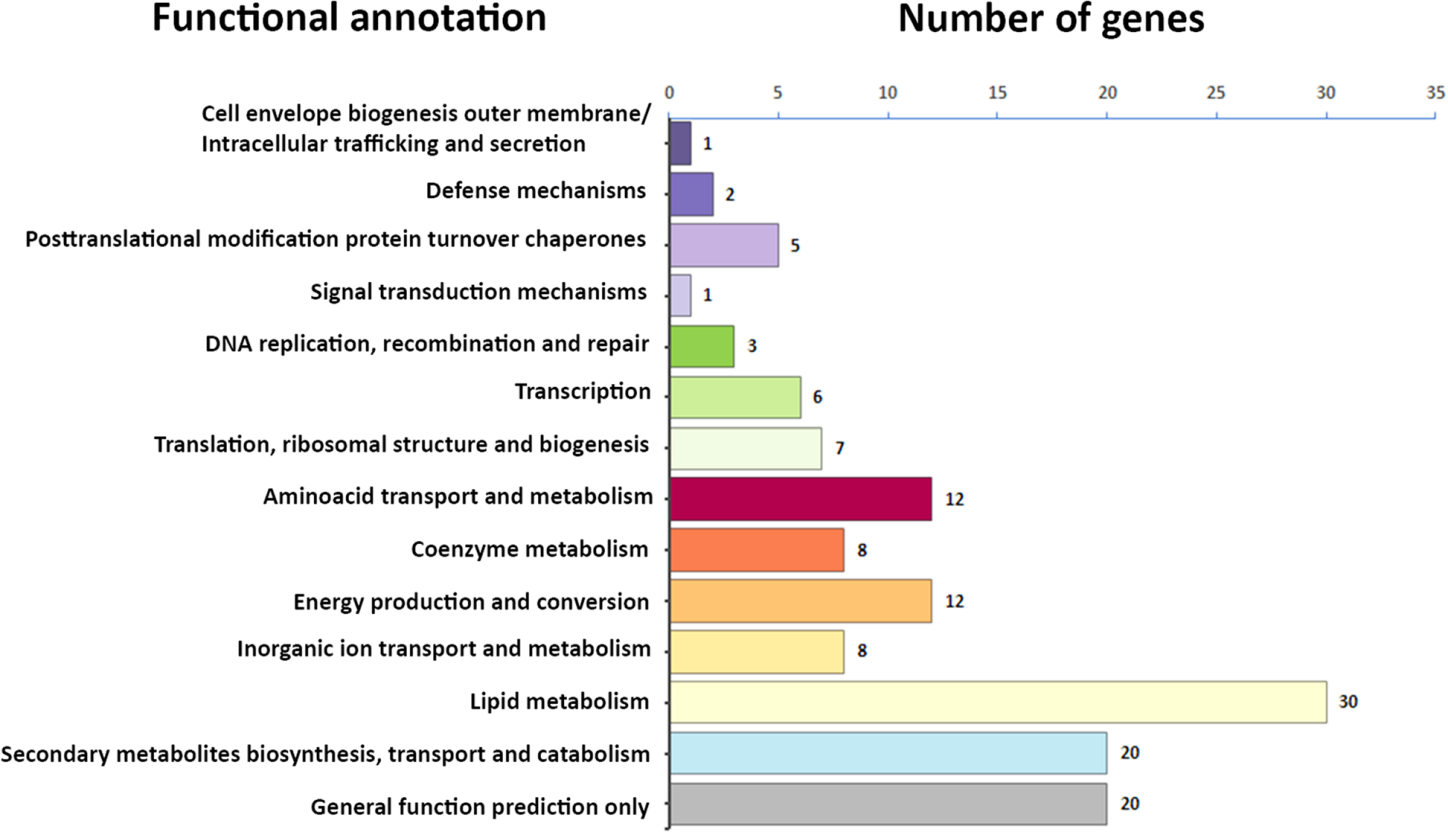
Functional annotation of the unique genes into 21 M*. tuberculosis* genome by eggNOG Mapper v2.1.7

Of particular interest are the strain-specific genes that may be responsible for the variability and particularities of this set. Therefore, based on the identification and classification of the genes, we performed a characterization analysis of the functional groups of strain-specific genes. By analyzing the distribution in the 21 strains, we found that most of the identified unique genes involved in metabolism and transport. In particular in the metabolism of lipids, secondary metabolites, coenzymes, amino acids, and inorganic compounds. A smaller proportion of genes are linked to replication, transcription, and translation, and only a few unique genes are related to cellular processes and signaling (Fig. 4).

Since our analysis is based on resistant strains, particular attention was devoted to specific genes, like those genes related to virulence. First, using the Virulence Factor Database (VFDB), a total of 64 virulence-related proteins implicated in the pathogenicity of *M. tuberculosis* were identified, classified as follows: 58.96% related to Secretion system; 11.36% to iron uptake, Siderophore; 4.26% to Regulation and Cellular Metabolism; 4.12% to the Cell wall and 1.42% to Heat-shock protein and Magnesium uptake (Supplementary table 4). Second, using DrugBank, 66 proteins were identified as potential drug targets (Supplementary table 5). Similarly, 38 families of Transporter proteins were identified by the Transporter Classification Database (TCDB), the most representative being the ATP-binding cassette (abc) superfamily (25.17%), followed by the type vii or esx protein secretion system (t7ss) family (23.65%), the general secretory pathway (sec) family (8.38%), the major facilitator superfamily (mfs) (5.86%) and the resistance-nodulation-cell division (rnd) superfamily (5.03%) (Supplementary table 6). Finally, the presence of CRISPR genes was analyzed using CRISPRfinder, and between 2 and 3 CRISPRs were detected in each genome (Supplementary table 1).

**Fig. 5:**
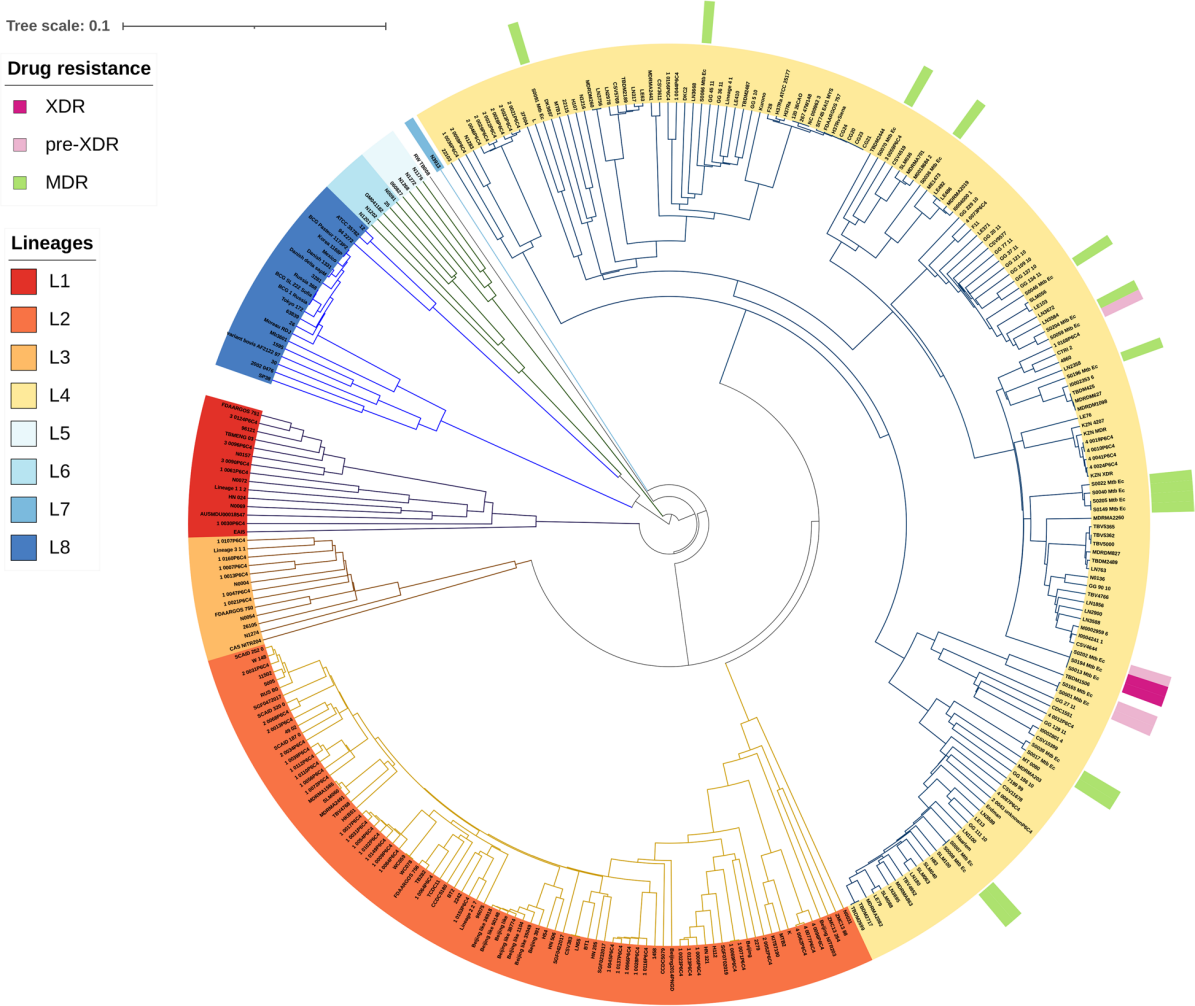
UPGMA tree for 21 Mtb genome analyzed in this study. The un-rooted tree was constructed based on 2891 core genes Multi Locus Sequence Typing (cgMLST) extracted from the 21 samples and 297 reference genomes using Ridom SeqSphere v8.3.1. Drug resistance of 21 Ecuadorian isolates are represented: XDR (Strong pink); pre-XDR (Very soft pink); MDR (Moderate green). Lineages were identified in all samples: L1 (Strong red); L2 (Bright red); L3 (Soft orange): L4 (Very soft orange); L5 (Light grayish cyan); L6 (Very soft blue); L7 (Slightly desaturated blue); L8 (Moderate blue). UPGMA = unweighted pair group method with arithmetic mean

**Fig. 6:**
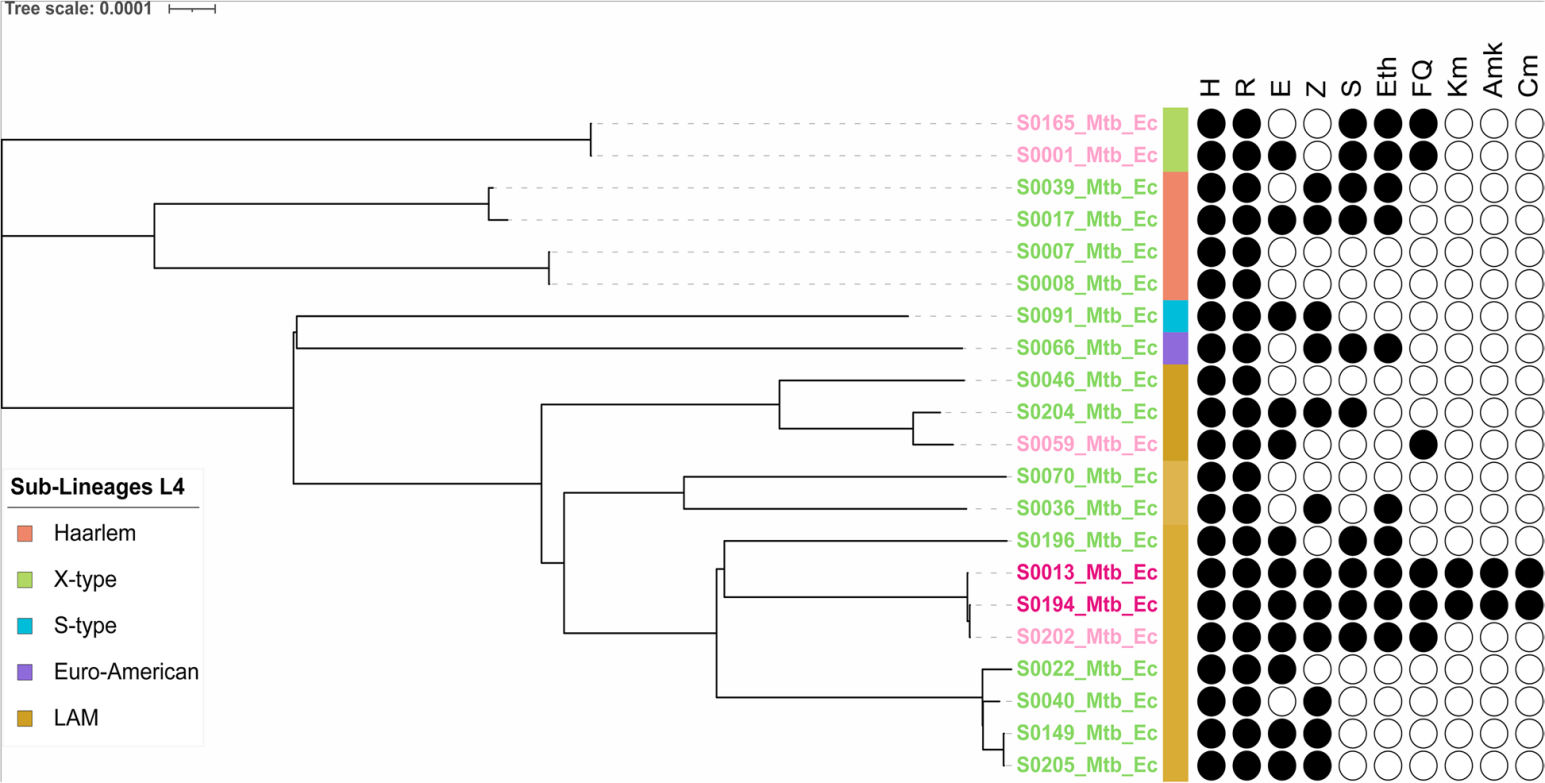
ML phylogenetic tree showing the relationship of 21 M*. tuberculosis* drug resistance pattern and Lineages. Abbreviations: rifampicin (R), isoniazid (H), pyrazinamide (Z), and ethambutol (E), fluoroquinolone (FQ), streptomycin (S), ethionamide (Eth), and aminoglycosides (Amk, Km, Cm). Color strips represent the sub-lineages. ML: Maximum Likelihood

From the genomes, in silico lineage inference was performed using different web tools: TB-Profiler v2.8.13, PhyReSse v1.0, SNP-IT, Mykrobe v0.10.0, CASTB v1.5, TB-Lineage, PhyTB, GenTB and a comparison of their performance was made. Moreover, in silico spoligotyping was determined by KvarQ v0.12. All genomes in this study belonged to Euro-American Lineage (Lineage 4), being the sub-lineages LAM (61.9%, 13/21) and Haarlem (19.0%, 4/21) the most representative, and clades X, S, and T founded in lowest percentage (Supplementary table 7 and Fig. 5). Among all lineages characterized by complete genomic sequencing, isolates classified as MDR accounted for 71.4% (15/21) and resistant (pre-XDR and XDR) for 28.6% (6/21). Notably, most of the latter belong to the LAM sub-lineage, and within the LAM sub-lineage, 30.8% (4/13) are resistant with great epidemiological importance (Fig. 6). TB-profiler, Mykrobe, PhyTB, and GenTB were the most informative web tools for lineages characterization. These tools detailed more genotypes with 100% concordance into *M. tuberculosis* sub-lineages. However, isolates from the T sub-lineage, including genotypes 4.8 to 4.10, could not be effectively resolved. PhyReSse, on the other hand, showed a lower ability to differentiate sub-genotypes within genotypes 4.1.2 and 4.3.4. TB-Lineage, SNP-IT, and CASTB could not effectively differentiate sub-lineages with a wide variety of genotypes and subgenotypes, especially the LAM sublineage (Table 2).

**Table 2:** Frequency and distribution of *Mycobacterium tuberculosis* sub-lineages in 21 Ecuadorian isolates characterized by web-tools

L4 Sub-lineages	Genotype	Web-tools							
		**PhyReSse**	**TB-profiler**	**Mykrobe**	**PhyTB**	**GenTB**	**TB-LINEAGE**	**SNP-IT**	**CASTB**
X-type/X3	4.1.1	2	2	2	2	2	2	2	2
Haarlem/H3	4.1.2	4	2	2	2	2	4	4	4
	4.1.2.1	0	2	2	2	2	0	0	0
LAM	4.3.2	3	3	3	3	3	13	13	13
	4.3.3	8	8	8	8	8	0	0	0
	4.3.4	2	0	0	0	0	0	0	0
	4.3.4.1	0	1	1	1	1	0	0	0
	4.3.4.2	0	1	1	1	1	0	0	0
S-type/H1	4.4.1	1	0	0	0	0	1	1	1
	4.4.1.1	0	1	1	1	1	0	0	0
Mainly T	4,8	1	1	0	1	1	0	0	0
	4,10	0	0	1	0	0	0	0	0
Lineage 4	ND	0	0	0	0	0	0	1	0
T1;T2;T3;T4	ND	0	0	0	0	0	1	0	1

*Abbreviations L4* Lineage 4, *ND* Not defined

To determine drug-resistance single nucleotide polymorphism (SNPs) and small insertions and deletion (indels), we performed a single nucleotide variant calling (SNV) comparing the samples with the H37Rv genome. From the 21 Mtb clinical isolates analyzed in this study, 21,596 high-quality variants were discovered (19,547 SNPs; 1154 insertions and 892 deletions) (Supplementary table 8). Overall, on average 1028 SNPs were found per sample (range 682–1152 SNPs), corresponding to a median SNP density of 1 SNP per 4.2 kb. Few variants were present in all samples, only 1.32% of SNPs (*n* = 289) and 0.8% of indels (*n* = 5). Most SNPs were found in coding regions (88%, 17,214) and the remaining 2,333 in intergenic regions. Of the SNPs in coding regions, the majority of the variants lead to non-synonymous (NS) changes in amino acids (*n* = 10,685), including modification of the coding gene (97.5%), loss of start codon (0.1%); gaining of stop codon (1.82%); lost of stop codon (0.6%). The amino acid changes more frequently were *Thr25Ala*, *Ile245Thr*, *Lys212Asn*, *Gly13Asp*, *Ala182Val*, *Asn140Ser*, *Gln20Leu*, *Gly33Ser*, *Thr136Ala*, and *Val259Ala* observed 27, 25, 24 23 and 22 each time, respectively. Among synonymous changes (6529) including 99.35% of synonymous, 0.4% of missense, 0.2% of stop retained variants.

**Fig. 7:**
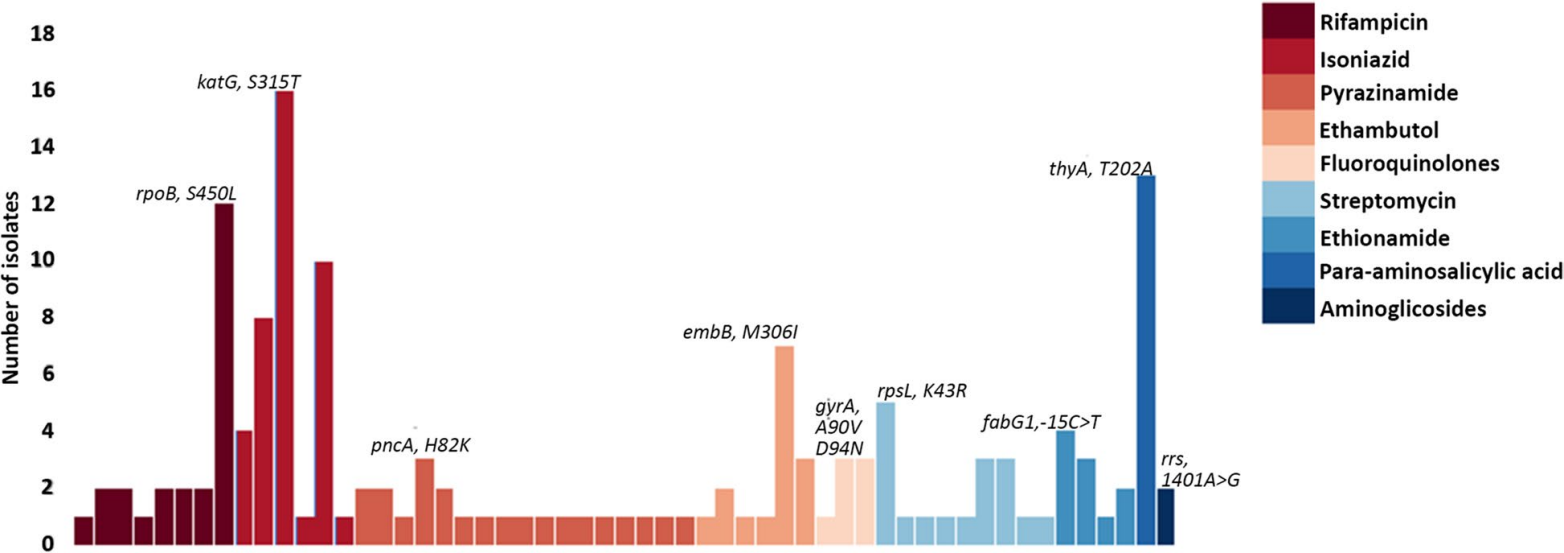
Frequency of SNVs known to confer resistance against first and second-line treatment drugs in 21 clinical isolates characterized by WGS

Applying the web-based tools TB-Profiler v2.8.13, PhyReSse v1.0 Mykrobe v0.10.0, CASTB, KvarQ v0.12, PhyTB, GenTB, SAM-TB for genomic analysis of 21 strains phenotypic characterized MDR and polydrug-resistance according to WHO guidelines we identified genotypically 71.43% of strains MDR (15/21), 19.05% pre-XDR (4/21) and 9.52% XDR (2/21). A total of 55 SNVs were identified and distributed into 18 genes known to confer resistance to first- and second-line drugs. Among these, 67.27% (37/55) SNVs were related to resistance to first-line drugs, and genetic regions associated with pyrazinamide resistance presented more frequent variation regions (30.9%, 17/55). Among all genomes, the most frequently genotype identified were *Ser315Thr* (76.2%,16/21), *Ser450Leu* (57.1%,12/21), and *Met306Ile* (33.3%, 7/21)*,* encoding a substitution in *katG, rpoB* and *embB* gene that confers resistance to rifampicin, isoniazid, and ethambutol, respectively; whereas for second-line drugs were *Thr202Ala* in *thyA* which is associated with para-aminosalicylic acid resistance in 61.9% (13/21) and *Lys43Arg* in *rpsL* related with streptomycin resistance (28.5%,6/21) (Fig. 7).

We also evaluated the prevalence of resistance in the fluoroquinolone among isolates sequenced by looking at *gyrA* and *gyrB* gene mutation frequencies. In the *gyrA* gene, the SNV *Asp94Asn*, known to confer fluoroquinolone resistance, was found in all pre-XDR isolates, while SNV *Arg90Val* was found in XDR isolate and one pre-XDR. Additionally, the known SNV *Ser95Thr* in the *gyrA* gene was found in 20 isolates. For resistance characterization in tuberculosis, the web-based tools SAM-TB (76.4%) and Mykrobe (72.7%) approached the most frequently identified mutations, complemented by PhyReSse (50.9%), to determine genetic resistance by whole-genome sequencing in surveillance programs (Supplementary table 9 and 10).

## Discussion

The present study describes for the first time the use of whole genome sequencing in the screening of TB drug susceptibility in Ecuador and provides information on the phylogenetic characteristics and the identification of the predominant mutations in circulating isolates. Despite its limited number of samples including in this study (24 isolates), it represents the initial effort to determine genetic diversity using WGS. Most isolates in our study were from Guayaquil (79.17%), one of the most economically important cities in Ecuador; the high number of tuberculosis cases detected in this city is possibly due to the high mobility from other provinces for trade or work in addition to the location of the leading health centers for the monitoring of this pathogen.

The potential of WGS to provide a quick and comprehensive view of the genotype and reliable prediction of the lineages has been extensively reported [5, 7, 14, 40]; thus, its application in surveillance programs is necessary. The Euro-American lineage (lineage 4) presents a variable distribution in Argentina with a predominance of M and Ra strains [41], for Brazil, lineage 4 varies between 25–99% [13, 42, 43], for Colombia, between 85–99% [44–46], for Mexico between 69–98% [47, 48] and for Peru around 90% [12, 49, 50]. Ecuador is not an exception, in fact, all the samples analyzed by us belong to the Euro-American lineage. This lineage in South and Central America and the Caribbean is represented mainly by the LAM, Haarlem, and T families sub-lineages [11, 41, 51]. In our study, most of the cases analyzed also corresponded to LAM (61.9%), followed by the Haarlem lineage (19.0%). This result provides genomic information from Ecuadorian isolates of *Mycobacterium tuberculosis* that contribute to the knowledge of the distribution of Mtb lineages in South America and confirms the European origin of the circulating strains in Ecuador [9, 52, 53]. Similar results have been described in some, but not all, previous studies using genotyping by 24-loci MIRU-VNTR, Spoligotyping, and SNP-PCR strategies in Ecuador [36, 54].

The movement or conservation of genes in bacteria is a fundamental factor related to virulence, survival, and host adaptability, allowing them to co-evolve together [55]. Several studies in *M. tuberculosis* have shown that the ability of transmission within populations, latency periods, and drug resistance are due to genetic differences within the members of the *M. tuberculosis* complex and that many of its genes have been under positive selection at different periods [56]. Furthermore, the composition within its genome remains relatively stable due to the absence of horizontal gene transfer, and this superficial level of genetic variation is probably because these pathogens have a strongly clonal nature [57]. The highly conserved genetic content and extreme clonal nature suggest that *M. tuberculosis* should have a relatively high percentage of core genome, however, analysis of core and pangenome have shown more variability than expected [58–61]. A pangenome is a union of the entire genetic pool of several strains of a species under comparison, essentially consisting of a core genome containing genes and sequences shared in all strains and an accessory genome consisting of genes and sequences which may be absent from one or more strains and genes that are unique to each strain remaining. In our study, the genetic composition of 21 drug-resistant isolates was analyzed, and 3750 genes (79.2%) were identified in the core genome, considering it a relatively large core genome in line with previous studies in which few genomes were evaluated. Therefore, it is necessary to expand the number of isolates to determine exactly which are the essential genes in *M. tuberculosis* within those circulating in Ecuador.

Characterizing the protein's functionality in microorganisms of importance in public health is essential to understanding their pathogenicity, resistance to antibiotics, and virulence, which allow them to adapt or survive in their host. Our results showed that annotations of protein-encoding genes are mainly associated with metabolisms being Amino acids and their derivatives, Energy production and conversion, and lipid metabolism, the most representative. Similar studies in other *M. tuberculosis* populations also showed a high presence of genes related to energy production and conversion (C), amino acid transport and metabolism (E), and lipid transport and metabolism (I) [72–74]. This suggests that these genes are widely conserved to ensure the interactions of *M. tuberculosis* with its human host, especially in mycobacterial persistence, host pathogens struggle for nutrients and immune recognition. On the other hand, we were able to identify mainly virulence factors associated with the secretion system (58.96%), the most important being the transporter proteins of the ATP-binding cassette (abc) superfamily (25.17%) and the type vii or esx protein secretion system (t7ss) family (23.65%). It has been suggested that high levels of transporters are involved in cell detoxification, nutrient recycling, and antibiotics and drug efflux, significantly affecting the survival and development of multiple drug-resistant strains in Mtb [58, 59, 62, 63]. It should be noted that the unique genes identified 2-C-methyl-D-erythritol 4-phosphate cytidylyltransferase and Arylamine N-acetyltransferase were previously characterized as high-confidence drug targets [64–66].

All resistant isolates analyzed showed any variations in genes related to confer resistance, classified them in 71.43% of strains MDR (15/21), 19.05% pre-XDR (4/21), and 9.52% XDR (2/21). Both XDR strains presented similar mutations profile (8 non-synonyms and one deletion SNV in the coding region, 2 SNV in the intergenic region), but differing in additional Ser315Gly mutation in the katG gene arise to isolate S0013_Mtb_Ec, for the four pre-XDR isolates the S0202_Mtb_Ec was that more mutations were identified (9 genomic regions). It is brought up that of these six isolates of concern, five belong to women within the same age range.

The study of the *M. tuberculosis* resistance is an important factor within its surveillance plan in all countries, for which the timely detection of strains resistant to treatment drugs allows the application of an efficient drug strategy, reducing or limiting the incidence of new cases, especially when applying culture-based methods to detect it. In this study, we found high concordance between WGS and conventional culture-based DST in predicting phenotypic drug resistance to anti-TB drugs; In addition, WGS was able to determine resistance patterns that DST does not evaluate. These findings are in agreement with previous studies [40, 67, 68]. Drug resistance is mediated through mutations in specific gene targets. The key, therefore, is to identify single nucleotide polymorphisms (SNPs) that are responsible for or strongly associated with resistance. In the case of the first-line drug in Mtb treatment, mutations in the 81-bp rifampicin resistance determining region (RRDR) of *the rpoB* gene, also known as a hotspot region, have been accurate predictors of rifampicin resistance in many studies [69–71]. On the other hand, it has been established that INH resistance, predominantly mediated through loss of catalase-peroxidase activity via mutations in katG, produces high-level resistant strains [18, 72, 73]. Finally, in ethambutol, most resistance-related genes are located on *the embB, embC*, and upstream of the embA [74, 75]. Meanwhile, previous studies have agreed that the genes *rrs*, *rpsL*, and *gid* are related to different levels of streptomycin resistance [76, 77], and the chromosomal mutations in the quinolone resistance determining region of *gyrA* or *gyrB* are the main mechanism of resistance to fluoroquinolones in *M. tuberculosis* [78, 79]*.* In our study, we found 57.1%, 70.0%, 47.6%, and 28.6% and 28.45% of mutations *S450L (rpoB), S315T/G (katG), M306V/I (embB), K43R* (*rpsL*) and c492t, a514c (*rrs* locus) related to rifampicin, isoniazid, ethambutol, streptomycin and fluoroquinolones resistance respectively. The increased resistance to fluoroquinolones, also recently reported in other studies [80], is of concern, probably caused by the increased administration of this drug without prescription [81]. It is therefore imperative to monitor the increase in these resistances in tuberculosis surveillance programs [82, 83].

Whole-genome sequencing (WGS) is a powerful method for detecting drug resistance, genetic diversity, and transmission dynamics of *M. tuberculosis*. Despite their advantages, the analysis of genomic sequencing data remains an obstacle to the routine use of WGS technology in clinical tuberculosis because it requires bioinformatics expertise, high-performance computing, funding, and training that are not readily available in most clinical laboratories [84, 85], which represents a significant challenge for TB control efforts. Over the last few years, many TB-specific genome browsers and WGS analysis tools such as TBProfiler, KvarQ, Mykrobe Predictor TB, CASTB, PhyTB, GenTB, PhyResSE, and others have been developed for genotyping and drug resistance identification, however, despite their use have not been widely used within antimicrobial resistance (AMR) surveillance program on low- and middle-income countries (LMICs) [86]. In our results, web-based tools enabled an effective and user-friendly identification of resistance-associated mutations in *M. tuberculosis*. The recently developed SAM-TB platform [21] is the most capable of determining the mutations present in the isolates of our study, which, when used in conjunction with web-based tools Mykrobe and PhyReSse, would allow rapid screening of *M tuberculosis* isolates. These tools could easily be implemented in surveillance programs based on microbiological procedures to obtain results efficiently and cheaply [84, 87, 88].

Given that the sample size used is small and could be underestimating the real frequencies of mutations in *Mycobacterium tuberculosis* present in this region, it becomes imperative to make greater efforts -and use the advantage of WGS- to analyze a larger number of samples to identify and study the mutations present in patients with tuberculosis including other regions of Ecuador.

## Conclusion

The findings of this study demonstrate the usefulness of applying next-generation sequencing tools such as WGS to characterize mutations and describe the existing variability in tuberculosis strains that allow adequate monitoring to generate health policies. We identified the variability of resistant strains circulating among men and women in Ecuador and showed that mainly strains of the American-European lineage 4 circulate, with a higher proportion of the LAM sub-lineage. XDR/MDR strains are not associated with a specific lineage, region or other metacharacter analyzed. However, we found an association between sex and resistance, which should be verified with further sampling. Within the observed resistances, an increase in fluoroquinolone resistance is evident, which should be monitored. Additional sampling is needed to determine the total variability and associations with the metadata obtained to generate better health policies.

Although *M. tuberculosis* is considered a highly conserved clonal species, among the 21 strains analyzed, we found unique genes specialized in lipid metabolism that are particularly interesting for identifying potential new drug targets.

## Methods

Twenty-four clinical isolates were sampled and collected from private laboratories between March to December 2020; these isolates had been previously characterized as multidrug-resistant through drug susceptibility testing. The resistance pattern were performed for first-line anti-tuberculosis drugs, isoniazid (H), rifampicin (R), pyrazinamide (Z), ethambutol (E), and one second-line drug (streptomycin (S)), the proportional method by [89] on Lowenstein-Jensen medium and the resistance profile was defined according to WHO recommendations. The critical concentrations of anti-TB drugs evaluated were as follows: rifampicin, 40.0 μg/ml; isoniazid, 0.2 μg/ml; ethambutol, 0.4 μg/ml; streptomycin, 4.0 μg/ml, and 200 µg/mL for pyrazinamide.

### DNA extraction and sequencing

Genomic DNA was extracted from 24 isolates of *Mycobacterium tuberculosis* grown in a Lowenstein-Jensen medium using the CTAB method [90]. The quantity of isolated DNA was measured using a Qubit 4.0 fluorometer (Invitrogen, Carlsbad, CA, USA.). DNA samples that fulfilled the integrity, purity, and quantity standards were sequenced. Genomic DNA libraries were prepared for whole genome sequencing using the Tagmentation-based library prep kit according to the manufacturer's instructions (Illumina Inc., San Diego, CA) and sequenced on an Illumina MiniSeq platform with High Output Reagent Kit, producing 150 bp paired-end reads.

### Bioinformatic analyses

Using the Galaxy platform (https://usegalaxy.org/), reads were classified by Kraken version 2 [91] to detect any contamination or the presence of other mycobacteria; In addition, FastQC version 0.11.9 [92] and Trimmomatic version 0.38 [93] were used to control the quality and trim the low-quality ends of the reads, respectively. In particular, a sliding window was used to trim sequences with an average quality value lower than 20.

### Genome assembly

The final high-quality reads were assembled using Megahit version 1.1.3 [94]. The assembly was carried out with a kmer range from 29 to 141 at intervals of 20 and 800 bp as minimum contig size. To perform contig taxonomic assignment, we used Kaiju version 1.9.0 [95, 96]. For generating metrics and evaluating the quality of the assemblies using *M. tuberculosis* strain HR37v (NC_000962) as a reference genome, Quast version 5.0.2 was used [97].

### Gene prediction and functional annotation

The structural and functional annotation was performed with Prokka version 1.12 [98] and the "Rapid Annotation using Subsystem Technology (RASTtk server)" pipeline online [38], and the predicted proteins were also annotated for the identification with the Clusters of Orthologous Groups (COG) by eggNOG Mapper v2.1.7 (http://eggnog-mapper.embl.de/). For the identification of CRISPRs, we used the webserver of CRISPRFinder (https://crispr.i2bc.paris-saclay.fr/Server/). Analysis of virulence-related proteins, potential drug targets, and Transporter proteins by Virulence Factor Database (VFDB), DrugBank, and Transporter Classification Database (TCDB) was performed in the Pathosystems Resource Integration Center (PATRIC) online (https://www.patricbrc.org/).

### Variant calling

Processed reads were mapped with Bowtie2 [99] using H37Rv (NCBI ID: NC_000962.3) as a reference genome. Sequence alignment files were sorted and indexed with Samtools v0.1.19 [100]. Bcftools [101] were used for calling variants, and vcftools [102] were used to filter the raw variants (minimum quality score of 30, minimum depth 10). From high-quality variants annotated for 21 genomes, the effect and impact were determined by SnpEff [103].

### Pangenome analyses

The pangenome was analyzed with Roary [37] and organized as follows: core, soft-core, shell, and cloud genes if the genes were presented in all, 20, 3—19, and 1—2 samples, respectively; the characteristic curves of the Mtb pangenome, the core-genome, and the new genes were depicted using the pangenome Profile Analyze Tool (PanGP) [58, 104, 105]. Alignments for the core genes Multi Locus Sequence Typing (cgMLST) were used for generating a phylogenetic tree using Ridom SeqSphere v8.3.1 [106]. The output tree was visualized and annotated using the online tool iTOL [107].

### Phylogenetic analysis

From high-quality reads the *M. tuberculosis* complex (MTBC) lineages/sublineages were determined and compared using the user-friendly web tools TB-Profiler v2.8.13 (https://tbdr.lshtm.ac.uk/), PhyReSse v1.0 (The Phylo-Resistance-Search-Engine) (https://bioinf.fz-borstel.de/mchips/phyresse/), SNP-IT [108], Mykrobe v0.10.0 (https://www.mykrobe.com/), CASTB v1.5 (The Comprehensive Analysis Server for the *Mycobacterium tuberculosis* complex) (http://castb.ri.ncgm.go.jp/CASTB/), TB-Lineage (https://tbinsight.cs.rpi.edu/run_tb_lineage.html), PhyTB (http://pathogenseq.lshtm.ac.uk/phytblive/index.php), GenTB (https://gentb.hms.harvard.edu/) and in silico spoligotyping were determined by KvarQ v0.12 (https://gap.tbportals.niaid.nih.gov/#/dashboard/home), the different web-programs were working on default parameters in our on-line pipeline.

### Predicting susceptibility and drug resistance

Genes related to resistance to rifampicin (R), isoniazid (H), pyrazinamide (Z), and ethambutol (E) [first-line drug], and fluoroquinolone (FQ), streptomycin (S), ethionamide (Eth), aminoglycosides (Amk, Km, Cm), para-aminosalicylic acid (PAS) [second-line drugs] were considered for the analysis. After variant calling and annotation, variants on each resistance gene were determined and compared from the variant call format (VCF) files using the web-based tools TB-Profiler v2.8.13, PhyReSse v1.0 Mykrobe v0.10.0, CASTB, KvarQ v0.12, PhyTB, GenTB, SAM-TB (https://samtb.szmbzx.com/index).

## Fundings

This work was funded by grant FCI-016–2017 from University of Guayaquil. The funders had no role in study design, data collection and analysis, decision to publish, or preparation of the manuscript. GM is a doctoral student in the PEDECIBA program. LB is a member of PEDECIBA and the Sistema Nacional de Investigadores (SNI) of ANII.

## Supplementary Information

**Additional file 1:** **Supplementary table 1.** Sociodemographic, epidemiological and assembly quality data from 21 isolates of Mycobacterium tuberculosis.

**Additional file 2: Supplementary table 2.** Distribution of COG annotations on 21 samples of Mycobacterium tuberculosis.

**Additional file 3: Supplementary table 3.** Distribution of Subsystems on 21 samples of Mycobacterium tuberculosis by Rastk.

**Additional file 4: Supplementary table 4.** Distribution of Virulence Factor on 21 samples of Mycobacterium tuberculosis.

**Additional file 5: Supplementary table 5.** Distribution of potential drug target on 21 samples of Mycobacterium tuberculosis.

**Additional file 6: Supplementary table 6.** Distribution of Transporters on 21 samples of Mycobacterium tuberculosis.

**Additional file 7: Supplementary table 7.** Characterization of Mycobacterium tuberculosis sub-lineages in 21 Ecuadorian isolates characterized by web-tools and their correlation with phenotypic and genotypic drug resistance profile.

**Additional file 8: Supplementary table 8.** Genetic variants characterized in 21 Ecuadorian isolates of Mycobacterium tuberculosis.

**Additional file 9: Supplementary table 9.** Mutations identified in resistance related genes by web-tools on 21 samples of Mycobacterium tuberculosis.

**Additional file 10: Supplementary table 10.** Distribution of mutations identified in resistance related genes for web-tools on 21 samples of Mycobacterium tuberculosis.

## Data Availability

The *Mycobacterium tuberculosis* whole-genome sequencing data will be deposited in the public archive of NCBI under the BioProject ID PRJNA827129, BioSamples SAMN29877831—SAMN29877851.

## Abbreviations

TB: Tuberculosis
Mtb: *Mycobacterium tuberculosis*
MTBC: *M. tuberculosis* Complex
R: Rifampicin
H: Isoniazid
Z: Pyrazinamide
E: Ethambutol
FQ: Fluoroquinolone
S: Streptomycin
Eth: Ethionamide
Amk, Km, Cm: Aminoglycosides
PAS: Para-aminosalicylic acid
MDR: Multi-drug resistance
XDR: Extreme-drug resistance
DST: Drug susceptibility testing
SNPs: Single nucleotide polymorphisms
Indels: Insertions, and deletions
WGS: Whole-genome sequencing
CDS: Coding sequences
VFDB: Virulence Factor Database
TCDB: Transporter Classification Database
cgMLST: core-genome Multi Locus Sequence Typing
COG: Clusters of Orthologous Groups
PATRIC: Pathosystems Resource Integration Center
